# Risk Stratification in Pulmonary Embolism: Prognostic Value of PESI, WELLS, PADUA, and IMPROVE Scores in Relation to Laboratory Markers

**DOI:** 10.3390/jcm15083141

**Published:** 2026-04-20

**Authors:** Daniela Nicoleta Crisan, Talida Georgiana Cut, Alexandra Herlo, Sorin Deacu, Lucian-Flavius Herlo, Nina Ivanovic, Lavinia Simona Neculai-Candea, Andreea Nelson Twakor, Gabriela-Florentina Țapoș, Raluca Dumache

**Affiliations:** 1 Doctoral School, “Victor Babes” University of Medicine and Pharmacy Timisoara, 300041 Timisoara, Romania; daniela.crisan@umft.ro (D.N.C.); flavius.herlo@umft.ro (L.-F.H.); nina.ivanovic@umft.ro (N.I.); gabriela.tapos@umft.ro (G.-F.Ț.); 2 Department XIII, Discipline of Infectious Diseases, “Victor Babes” University of Medicine and Pharmacy Timisoara, E. Murgu Square, Nr. 2, 300041 Timisoara, Romania; talida.cut@umft.ro; 3 Department of Forensic Medicine, “Sf. Apostol Andrei” Emergency County Hospital, 900439 Constanta, Romania; lavinia.candea@365.univ-ovidius.ro; 4 Department of Dermatology, “Victor Babes” University of Medicine and Pharmacy Timisoara, E. Murgu Square, Nr. 2, 300041 Timisoara, Romania; 5 Department of Internal Medicine, “Sf. Apostol Andrei” Emergency County Hospital, 145 Tomis Blvd., 900591 Constanta, Romania; andreea.purcaru@365.univ-ovidius.ro; 6 Department of Forensic Medicine, Bioethics, Medical Ethics and Medical Law, “Victor Babes” University of Medicine and Pharmacy Timisoara, 300041 Timisoara, Romania; raluca.dumache@umft.ro; 7 Center for Ethics in Human Genetic Identifications, “Victor Babes” University of Medicine and Pharmacy Timisoara, E. Murgu Square, Nr. 2, 300041 Timisoara, Romania

**Keywords:** pulmonary embolism, risk stratification, Pulmonary Embolism Severity Index, WELLS score, PADUA score, IMPROVE score, in-hospital mortality prediction, thromboembolism, biomarkers, logistic regression

## Abstract

**Background**: Pulmonary embolism (PE) is a major cause of morbidity and in-hospital mortality and requires rapid clinical assessment for diagnosis, risk stratification, and management. Several validated clinical prediction tools, including the Pulmonary Embolism Severity Index (PESI), WELLS, PADUA, and IMPROVE scores, are commonly used to evaluate thromboembolic risk and predict clinical outcomes. **Methods**: A retrospective observational study was conducted on a cohort of 538 patients diagnosed with PE, all recruited between January 2020–December 2025. Group comparisons between survivors and non-survivors were performed using independent samples *t*-tests and Mann–Whitney U tests. Receiver operating characteristic (ROC) curve analysis was used to evaluate the predictive performance of clinical scores. Multivariable logistic regression analysis was performed to identify independent predictors of in-hospital mortality. **Results**: Mean age was 69 years, and overall death-rate was 18.4%. Significant differences between survivors and non-survivors were observed for age and clinical scores. White blood cell count, neutrophils, lymphocytes, platelet count, total bilirubin, aspartate aminotransferase (AST), alanine aminotransferase (ALT), gamma-glutamyl transferase (GGT), procalcitonin, and international normalized ratio (INR), were significantly associated with in-hospital mortality. ROC curve analysis demonstrated predictive performance of the evaluated clinical scores. Logistic regression identified PESI score, procalcitonin levels, and white blood cell count as independent predictors of unfavorable outcome. mortality. **Conclusions**: Clinical risk scores and specific laboratory biomarkers were associated with in-hospital mortality in patients with pulmonary embolism. The PESI score, procalcitonin, and white blood cell count showed independent predictive value for death rate in this cohort.

## 1. Introduction

Pulmonary embolism is a potentially life-threatening cardiovascular condition caused by obstruction of the pulmonary arterial circulation, most commonly due to thrombi originating from deep veins of the lower extremities. Venous thromboembolism, which includes deep vein thrombosis and pulmonary embolism, represents a major cause of morbidity and mortality worldwide and is recognized as one of the most frequent cardiovascular emergencies encountered in clinical practice [1].

In recent years, there has been growing interest in the role of laboratory biomarkers in improving prognostic assessment in PE. Biomarkers such as procalcitonin and white blood cell count show underlying inflammatory and systemic responses, which have been associated with disease severity and adverse clinical outcomes [2]. The physiological importance and regulation of PCT production is not well understood. Several hypotheses suggest that PCT may be involved in the metabolism of calcium, the cytokine network, and the modulation of nitric oxide synthesis, as well as having pain-relieving effects [3]. A Cochrane systematic review of 26 randomized controlled trials found that PCT-guided therapy for acute respiratory tract infections resulted in a significantly lower mortality rate, was linked to a 2.4-day decrease in antibiotic exposure, and resulted in a reduced risk for antibiotic-associated side effects [4].

The clinical spectrum of pulmonary embolism ranges from asymptomatic or mildly symptomatic cases to severe presentations characterized by hemodynamic instability, obstructive shock, or sudden cardiac death [3].

The Global Burden of Disease studies have not yet provided specific estimates for quality-adjusted life years (QALYs) or disability weights (used in calculating DALYs) for venous thromboembolism or pulmonary embolism [5].

PE represents the third most common cause of cardiovascular mortality, following coronary artery disease and stroke. If left untreated, PE is associated with a 30-day mortality rate of approximately 30%, while nearly 11% of patients may die within the first hour after hospital presentation [4]. Beyond the acute phase, venous thromboembolism (VTE) is associated with significant long-term morbidity. Post-thrombotic syndrome develops in approximately 20–30% of patients with deep vein thrombosis (DVT), while chronic thromboembolic pulmonary hypertension occurs in 1–3% of individuals following acute PE. Additionally, up to half of patients may experience persistent exercise limitation, and recurrence rates remain substantial, with nearly one-third of patients developing recurrent DVT or PE within 10 years [6].

The diagnosis and management of pulmonary embolism rely on a combination of clinical assessment, imaging techniques, and laboratory investigations. Modern diagnostic algorithms frequently incorporate computed tomography pulmonary angiography, which has become the imaging modality of choice for confirming the presence of pulmonary arterial obstruction [2]. In addition to imaging, laboratory markers such as D-dimer and inflammatory parameters are commonly used to support diagnostic evaluation and assess disease severity [4].

Thrombolytic therapy represents a therapeutic strategy used to restore pulmonary arterial blood flow in patients with pulmonary embolism by promoting the dissolution of intravascular thrombi. In pulmonary embolism, thrombotic material obstructs the pulmonary artery or its branches, leading to impaired blood flow, increased pulmonary vascular resistance, and potential hemodynamic compromise [5]. Thrombolytic agents act by activating fibrinolytic pathways, resulting in the degradation of fibrin within the thrombus and progressive fragmentation of the clot.

Risk stratification plays a fundamental role in the management of pulmonary embolism. Several prognostic models and clinical scoring systems have been developed to categorize patients according to their risk of adverse outcomes. These tools integrate clinical variables, comorbid conditions, and physiological parameters to identify patients at low, intermediate, or high risk of mortality or clinical deterioration [4]. Contemporary research also emphasizes the importance of combining clinical scores with laboratory markers to improve prognostic assessment and guide therapeutic decision-making in patients with pulmonary embolism [7].

Although several clinical risk scores have been developed to stratify patients with PE, including the PESI, WELLS score, PADUA prediction score, and IMPROVE score, these tools are often used in isolation and primarily focus on clinical variables [8]. The integration of these scoring systems with laboratory biomarkers remains insufficiently explored in real-world settings. This represents an important clinical gap, as a combined approach may provide a more comprehensive and accurate assessment of patient prognosis.

The selection of the PESI, WELLS, PADUA, and IMPROVE scores over other models like the Geneva score is based on their superior evidence-based validation, high clinical specificity for ruling out disease, and integration into international, evidence-based guidelines for VTE and PE management. These tools are recognized for accurately identifying low-risk patients suitable for outpatient management or for preventing unnecessary thromboprophylaxis in low-risk individuals. PESI was selected for mortality risk stratification, WELLS for diagnostic probability, PADUA for thrombotic risk assessment, and IMPROVE for risk evaluation in hospitalized patients [8].

**Figure 1:**
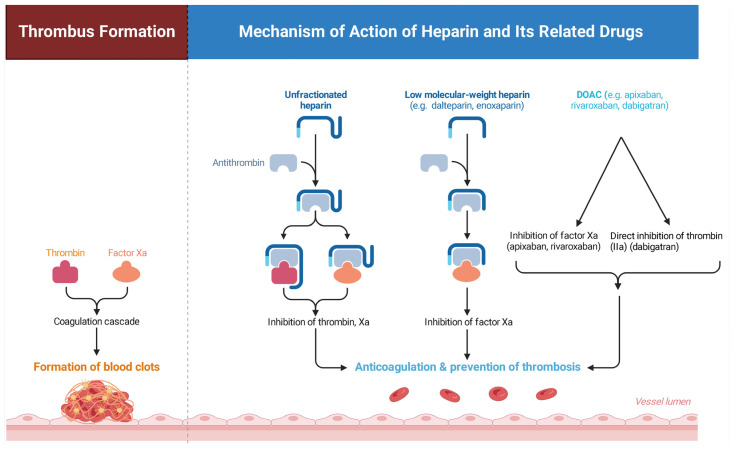
Mechanism of action of heparin and DOACs in the prevention of thrombus formation. Created in BioRender. Twakor, A. (2026) https://BioRender.com/pb0r9uv [11].

Low molecular-weight heparins (LMWH), including agents such as dalteparin and enoxaparin, are widely used anticoagulant medications for the prevention and treatment of thromboembolic disorders [9]. Their anticoagulant activity is mediated through interaction with antithrombin, a naturally occurring inhibitor of several activated coagulation factors. LMWH interferes with key steps of the coagulation cascade responsible for thrombin generation and fibrin clot formation by enhancing the inhibitory effect of antithrombin on factor Xa [10]. The schematic illustration in Figure 1 presents the relationship between thrombus formation through the coagulation cascade and the anticoagulant mechanism through which heparin and DOACs prevent the progression of thrombosis.

Activation of the coagulation cascade involves the interaction of thrombin and coagulation factors, leading to the formation of fibrin and the development of a blood clot along the vascular wall [10]. This process results in the accumulation of fibrin strands, platelets, and red blood cells, ultimately producing a thrombus within the vessel lumen. The right side of the figure demonstrates the anticoagulant mechanism of low molecular-weight heparin. LMWH binds to antithrombin and enhances its inhibitory activity against activated factor Xa [12]. The inhibition of factor Xa reduces thrombin generation and interrupts the progression of the coagulation cascade. As a result, clot formation is prevented and normal blood flow within the vessel is maintained, illustrating the role of LMWH in anticoagulation and thrombosis prevention [13].

The PESI [14], WELLS score [15], PADUA prediction score [16], and IMPROVE score [17] are clinical risk assessment tools used to evaluate thromboembolic risk, disease severity, and clinical outcomes in patients with suspected or confirmed venous thromboembolism. The PESI score is designed to estimate mortality risk in patients with pulmonary embolism by incorporating clinical variables such as age, comorbidities, vital signs, and oxygen saturation, allowing stratification into risk classes associated with short-term mortality [18,19]. The WELLS score is a clinical prediction rule used to estimate the pre-test probability of pulmonary embolism based on clinical findings including signs of deep vein thrombosis, heart rate, recent surgery or immobilization, previous thromboembolism, hemoptysis, malignancy, and clinical judgment regarding alternative diagnoses [20]. The PADUA prediction score is a validated risk assessment model used primarily in hospitalized medical patients to identify the risk of venous thromboembolism by evaluating factors such as active cancer, reduced mobility, previous thromboembolism, thrombophilia, trauma or surgery, and advanced age [21]. The IMPROVE score is another clinical model developed to assess the risk of venous thromboembolism in hospitalized patients by integrating multiple clinical variables including previous VTE, known thrombophilia, immobilization, intensive care unit stay, and other risk conditions [22,23].

The aim of this study was to evaluate the prognostic value of integrating clinical risk scores (PESI, WELLS, PADUA, and IMPROVE) with selected laboratory biomarkers in patients with pulmonary embolism.

We hypothesized that a combined approach incorporating both clinical scores and laboratory parameters would improve the prediction of in-hospital mortality compared to the use of individual scoring systems alone.

## 2. Materials and Methods

### 2.1. Study Design and Population

**Figure 2:**
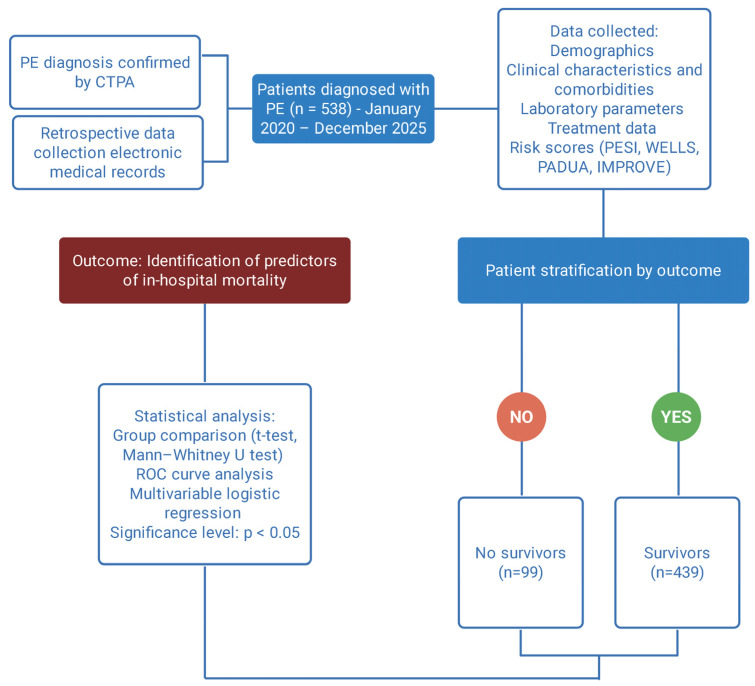
Study design and analytical workflow of the retrospective cohort. Created with Biorender [11].

This retrospective observational study included patients diagnosed with pulmonary embolism who were admitted to Arad County Clinical Hospital, Romania. A total of 538 patients recruited between January 2020–December 2025 were included in the study cohort (Figure 2). The diagnosis of pulmonary embolism was confirmed by computed tomography pulmonary angiography (CTPA), defined by the presence of an intraluminal filling defect in the pulmonary arterial tree at the level of the main, lobar, segmental, or subsegmental arteries. Key criteria include a partial or complete vessel obstruction, often showing a “polo mint” (short-axis) or “railway track” (long-axis) sign, where contrast surrounds the thrombus. An eccentric or mural filling defect rendering an acute angle with a vessel wall or complete occlusion of a dilated vessel by a filling defect can also be seen. This manuscript was designed as a retrospective observational analysis, and all consecutive patients meeting the inclusion criteria during the study period were included. Therefore, no a priori sample size calculation was performed. The study was conducted in accordance with the Declaration of Helsinki and was approved by the Ethics Committee of Victor Babes University of Medicine and Pharmacy Timisoara (approval number: 39; date: 3 March 2025).

Patient data were extracted from institutional electronic medical records and anonymized before analysis. The study population included both male and female patients with varying clinical presentations and comorbid conditions. Demographic characteristics, clinical variables, imaging findings, laboratory parameters, and therapeutic interventions were recorded for each patient. No formal exclusion criteria were applied, except for cases with incomplete or missing essential clinical or outcome data.

### 2.2. Data Collection

Clinical and demographic variables collected included age, sex, place of residence (urban or rural), thrombus localization, and the presence of comorbidities such as diabetes, cancer, obesity, pulmonary hypertension, and cardiac failure. Information regarding respiratory support and intensive care interventions, including intubation and continuous positive airway pressure (CPAP), was also documented. Laboratory parameters were collected at admission, defined as within the first 24 h of hospital presentation. They included white blood cell count (WBC), neutrophils, lymphocytes, platelet count, creatinine, total bilirubin, liver enzymes (AST, ALT, GGT), procalcitonin, D-dimers, activated partial thromboplastin time (aPTT), international normalized ratio (INR), and serum electrolytes (sodium and potassium). Serum samples were collected and sent for analysis of IL-6, CRP, D-dimers and fibrinogen. The analysis was performed on Multiskan FC Microplate Photometer (ThermoScientific, Waltham, MA, USA) using the ELISA sandwich (enzyme-linked immunosorbent assay) principle. IL-6 ELISA Human kit (Invitrogen, Carlsbad, CA, USA), CRP Human ELISA kit (Invitrogen, USA) and D-Dimer Human ELISA Kit (Invitrogen, USA) were used for the analysis. Biosafety Level 3 laboratory practices were adhered to. Expectorate sputum was selected as the sampling technique and routine microbiological investigations were conducted at the medical microbiology laboratory using standard bacteriology. All isolates were first identified using the VITEK^®^ 2 GN and VITEK^®^ 2 GP ID cards (BioMérieux, Marcy, l’Etoile, France). Antimicrobial susceptibility tests were performed using the VITEK 2 GN AST-N222 and VITEK 2 AST GP 67 cards (BioMérieux, Marcy, l’Etoile, France).

Data regarding pharmacological treatment, including thrombolytic therapy and anticoagulant medications such as enoxaparin, fondaparinux, unfractionated heparin, nadroparin, and vitamin K antagonists, were also collected.

### 2.3. Clinical Risk Scores

For each patient, several validated clinical scoring systems were calculated in order to evaluate thromboembolic risk and disease severity. These included the PESI, the WELLS score, the PADUA prediction score, and the IMPROVE score. These scoring systems incorporate clinical variables such as patient age, comorbidities, vital signs, and risk factors associated with venous thromboembolism. They were used to assess risk stratification and to analyze their association with clinical outcomes, particularly in-hospital mortality. Clinical scores were calculated for each patient based on standard published criteria, using clinical, demographic, and laboratory variables recorded at admission.

### 2.4. Statistical Analysis

Statistical analysis was performed using IBM SPSS Statistics (Version 30.0, IBM Corp., Armonk, NY, USA) [24]. Descriptive statistics were used to summarize baseline demographic and clinical characteristics. Continuous variables were reported as mean, standard deviation, median, minimum, and maximum values, while categorical variables were expressed as frequencies and percentages with corresponding 95% confidence intervals. Comparisons between survivors and non-survivors were performed using independent samples *t*-tests for normally distributed variables and Mann–Whitney U tests for non-normally distributed variables. ROC curve analysis was used to evaluate the predictive performance of the clinical risk scores for in-hospital mortality. Multivariate logistic regression analysis was conducted to identify independent predictors of death-rate. A *p*-value < 0.05 was considered statistically significant. The distribution of continuous variables was further assessed using normality tests (Kolmogorov–Smirnov and Shapiro–Wilk), the results of which are presented in Supplementary Table S1. The study dataset was complete for all included patients, and all variables required for the analyses were available.

## 3. Results

Table 1 summarizes the baseline demographic, clinical, and therapeutic characteristics of the patients included in the study cohort. Demographic data, thrombus localization, comorbidities, and the use of antithrombotic or supportive therapies were recorded in order to characterize the population and provide the clinical context necessary for subsequent risk stratification analyses. The frequencies, proportions, and corresponding 95% confidence intervals are presented for each variable to provide a precise estimation of the distribution of clinical characteristics within the cohort.

The study population showed a balanced sex distribution, with 265 males (49.3%) and 273 females (50.7%). Most patients originated from urban areas (58.6%), while 41.4% were from rural regions. Overall, in-hospital mortality in the cohort was 18.4%, with 99 recorded deaths. Regarding thrombus localization, lobar (29.7%) and segmental (29.1%) arteries were the most frequent sites of embolism, followed by the main pulmonary artery (25.7%). Subsegmental involvement was less frequent (3.7%), while thrombi located in the pulmonary trunk accounted for 3.9% of cases. Antiplatelet therapy was administered in 22.11% of patients.

Cardiac failure was present in varying degrees, with NYHA class II representing the most common category (36.2%), followed by class 0 (34.2%) and class III (15.6%). Pulmonary hypertension was documented in 43.5% of patients. Among comorbid conditions, diabetes was present in 22.9% of cases, cancer in 19.9%, and obesity in 31.2%. Sepsis was identified in 13.9% of patients, while associated infections were recorded in 44.98%. Regarding therapeutic strategies, alteplase was administered in 8.9% of patients, nadroparin in 10.96%, and unfractionated heparin in 11.0%. Vitamin K antagonists were used in 6.9% of cases, enoxaparin in 36.4%, and fondaparinux represented the most frequently administered anticoagulant (77.7%).

Comorbid conditions were present in the majority of the cohort (82.7%). Respiratory support measures included endotracheal intubation in 17.5% of patients and CPAP ventilation in 12.8%. Additionally, 23.2% of patients had a confirmed COVID-19 infection during hospitalization.

Table 2 presents the distribution of biological parameters and clinical risk scores recorded in the study cohort. These variables were collected at admission and reflect both the physiological status of patients and the severity of pulmonary embolism. Continuous variables are expressed as mean and standard deviation, together with 95% confidence intervals, median values, and observed ranges (minimum–maximum). The laboratory markers include hematological, inflammatory, hepatic, renal, and coagulation parameters, while the clinical section summarizes the principal prognostic scores commonly used in pulmonary embolism risk stratification, including PESI, WELLS, PADUA, and IMPROVE.

The mean age of the study population was 69 ± 14 years, with a median age of 70 years and a range between 20 and 95 years. Regarding hematological parameters, the mean white blood cell count was 10 ± 5 × 10^9^/L, while neutrophils averaged 10 ± 12 × 10^9^/L and lymphocytes 2 ± 2 × 10^9^/L. Platelet counts showed a mean value of 231 ± 97 × 10^9^/L. Renal function markers indicated a mean creatinine level of 1.36 ± 4.06 mg/dL. Liver function tests revealed mean values of 0.77 ± 0.78 mg/dL for total bilirubin, 67 ± 233 U/L for AST, 67 ± 223 U/L for ALT, and 71 ± 72 U/L for GGT.

Inflammatory activity was reflected by a mean procalcitonin level of 1.17 ± 3.58 ng/mL. Coagulation markers showed a mean D-dimer level of 10 ± 25 units, an aPTT of 26.0 ± 8.4 s, and an INR of 1.16 ± 0.33. Electrolyte values were relatively stable, with mean sodium levels of 139 ± 5 mmol/L and potassium levels of 4.1 ± 0.6 mmol/L. Regarding clinical risk scores, the mean PESI score was 110 ± 33, while the WELLS score averaged 1.8 ± 2.0. The PADUA score showed a mean value of 3 ± 2, and the IMPROVE score had a mean of 3 ± 2.

The comparative analysis of baseline demographic and clinical characteristics between survivors and non-survivors revealed several notable differences associated with in-hospital mortality (Table 3).

Female patients exhibited a higher death-rate compared to males (20.9% vs. 15.8%). Patients from rural areas had a greater proportion of deaths compared to those from urban areas (25.1% vs. 13.7%). With regard to thrombus localization, segmental artery involvement was associated with the highest mortality rate (23.7%), whereas subsegmental embolism showed the lowest in-hospital mortality (10.0%).

All evaluated clinical risk scores were consistently higher in non-survivors, with mean values of 126 vs. 106 for PESI, 2.5 vs. 1.7 for WELLS, 4 vs. 3 for PADUA, and 4 vs. 2 for IMPROVE, indicating a clear association between increasing score values and in-hospital mortality risk. Among comorbidities, advanced cardiac failure demonstrated a progressive increase in unfavorable outcome, with NYHA class IV showing the highest rate (46.2%). Sepsis had the most pronounced impact, with an in-hospital mortality rate of 60.0% compared to 11.7% in patients without sepsis.

Therapeutic and supportive interventions were also associated with outcomes. Patients receiving anticoagulant therapy had higher in-hospital mortality (25.3% vs. 15.5%), likely reflecting greater baseline severity. Similarly, the need for respiratory support was strongly associated with poor prognosis, with fatality rates of 46.4% in patients requiring CPAP and 48.9% in those requiring intubation.

In contrast, certain conditions such as pulmonary hypertension and obesity were associated with lower observed mortality rates (10.3% vs. 24.7% and 9.5% vs. 22.4%, respectively). Additionally, patients with COVID-19 infection had higher in-hospital mortality compared to non-infected individuals (28.8% vs. 15.3%).

To further evaluate the relationship between established clinical risk scores and in-hospital mortality, a comparative analysis was performed between survivors and non-survivors (Table 4). Continuous variables were analyzed using the independent samples *t*-test, with the appropriate variance assumption selected based on Levene’s test for equality of variances. The analysis included age and the main clinical risk scores used in pulmonary embolism assessment.

Significant differences were observed between survivors and non-survivors across all evaluated clinical scores. Age differed significantly between the two groups (t = −2.36, *p* = 0.019), indicating that patients who died were older on average compared with survivors. The PESI score showed a marked difference between groups (t = −4.72, *p* < 0.001), with higher values observed in patients who died. Similarly, the WELLS score was significantly higher in non-survivors (t = −3.37, *p* < 0.001). The PADUA score also demonstrated a significant difference between groups (t = −4.83, *p* < 0.001). The IMPROVE score exhibited the strongest association with death (t = −5.86, *p* < 0.001).

The distribution characteristics of age and clinical scores across outcome groups are illustrated in the Supplementary Materials (Figures S5–S12), providing additional insight into data dispersion and group differences.

To further investigate the relationship between biological markers and clinical outcomes, a non-parametric analysis was performed comparing laboratory parameters between survivors and non-survivors (Table 5). Because most biochemical variables showed non-normal distributions, the Mann–Whitney U test was applied to assess differences between the two independent groups defined by in-hospital mortality. The analysis included inflammatory markers, hematological indices, hepatic function parameters, and coagulation markers.

Several laboratory parameters demonstrated statistically significant differences between survivors and non-survivors. White blood cell count (Z = −4.947, *p* < 0.001), neutrophil count (Z = −5.603, *p* < 0.001), and lymphocyte count (Z = −4.049, *p* < 0.001) were significantly associated with in-hospital mortality. Platelet levels also differed between groups (Z = −2.154, *p* = 0.031). Liver function markers including total bilirubin (Z = −2.794, *p* = 0.005), AST (Z = −4.188, *p* < 0.001), ALT (Z = −1.992, *p* = 0.046), and GGT (Z = −2.894, *p* = 0.004) were significantly elevated among patients who died. Inflammatory activity was further reflected by significantly higher procalcitonin levels in non-survivors (Z = −4.502, *p* < 0.001). Coagulation imbalance was also observed, as INR showed a significant association with in-hospital mortality (Z = −3.830, *p* < 0.001). In contrast, no statistically significant differences were observed for creatinine (*p* = 0.400), D-dimer (*p* = 0.664), aPTT (*p* = 0.423), sodium (*p* = 0.228), or potassium (*p* = 0.462).

**Figure 3:**
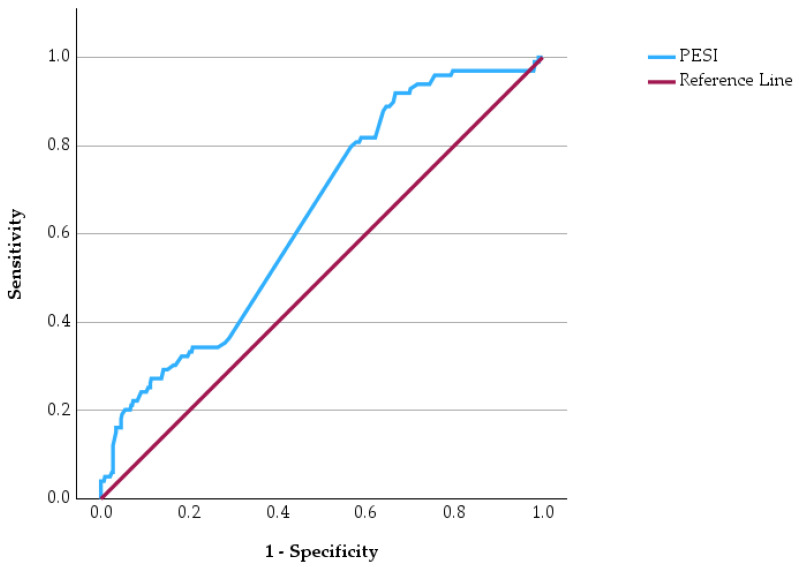
ROC curve of the PESI score for predicting in-hospital mortality.

**Figure 4:**
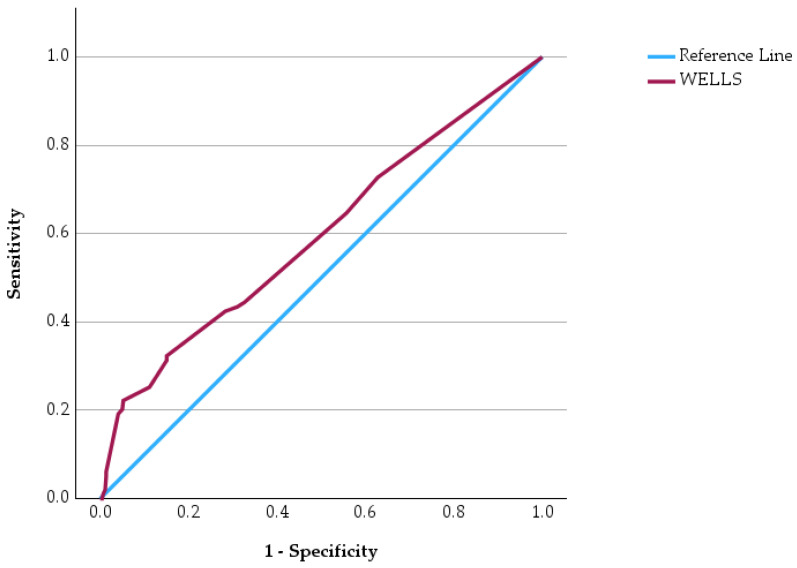
ROC curve of the WELLS score for predicting in-hospital mortality.

**Figure 5:**
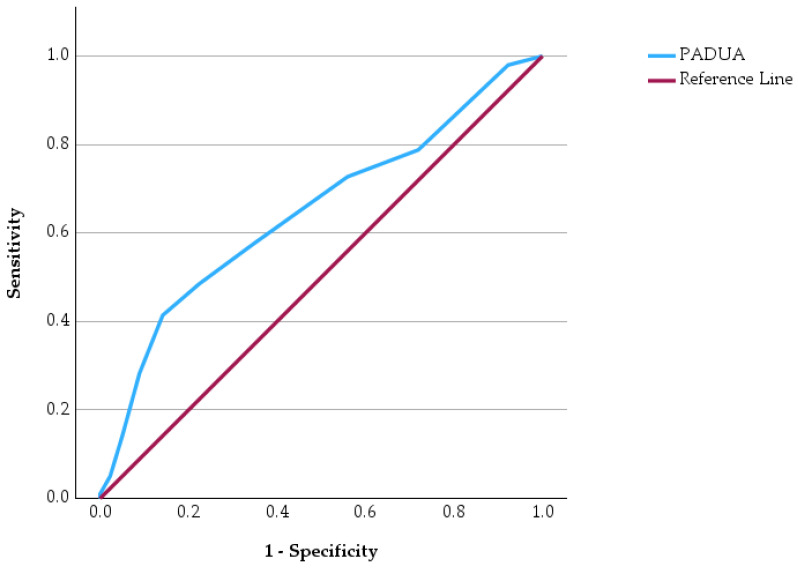
ROC curve of the PADUA score for predicting in-hospital mortality.

**Figure 6:**
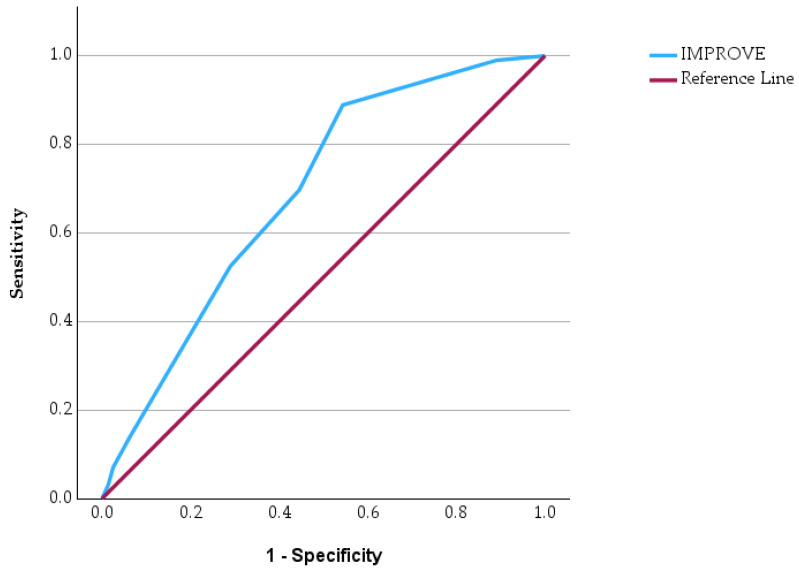
ROC curve of the IMPROVE score for predicting in-hospital mortality.

Receiver operating characteristic (ROC) curve analysis was performed to evaluate the predictive performance of four commonly used clinical risk score in identifying patients at increased risk of in-hospital mortality (Figure 3, Figure 4, Figure 5 and Figure 6). ROC curves has allowed us to carry out the assessment of the discriminatory ability of a prognostic tool by examining the relationship between sensitivity and specificity across different threshold values. In the graphical representation, the diagonal reference line indicates the absence of predictive capacity, whereas curves positioned above this line reflect increasing discriminatory performance. In addition to ROC analysis, precision–recall curves were generated for each clinical score to further evaluate predictive performance in the context of class imbalance (Figures S1–S4, Supplementary Materials).

The ROC curves demonstrate varying predictive performances among the evaluated clinical scores. The PESI score shows the most pronounced deviation above the reference line, indicating a stronger ability to discriminate between survivors and non-survivors. The IMPROVE score also demonstrates a noticeable upward trajectory, suggesting a meaningful predictive capacity for unfavorable outcomes. In contrast, the WELLS score shows a more modest separation from the reference line, indicating a weaker but still present discriminatory ability. Similarly, the PADUA score displays a moderate deviation from the diagonal, reflecting limited predictive strength.

A multivariate logistic regression analysis was performed to identify independent predictors of in-hospital mortality. Variables that were clinically relevant and previously associated with death rate in univariate analyses were included in the model. The predictors entered in the regression included age, the PESI score, and selected laboratory markers reflecting inflammatory and coagulation status, namely procalcitonin, WBC, and INR. Odds ratios (Exp(B)) with corresponding 95% confidence intervals were calculated to quantify the strength of the associations between these variables and death risk (Table 6).

The multivariate analysis identified several independent predictors of in-hospital mortality. The PESI score was significantly associated with death (OR = 1.013, 95% CI: 1.006–1.021, *p* < 0.001), indicating that higher PESI values were linked to an increased risk of death. Procalcitonin was also identified as an independent predictor (OR = 1.107, 95% CI: 1.028–1.191, *p* = 0.007), suggesting that elevated inflammatory activity was associated with worse outcomes. Similarly, WBC count showed a strong independent association with mortality (OR = 1.102, 95% CI: 1.056–1.149, *p* < 0.001). In contrast, age (*p* = 0.212) and INR (*p* = 0.649) were not significantly associated with mortality in the multivariate model.

**Figure 7:**
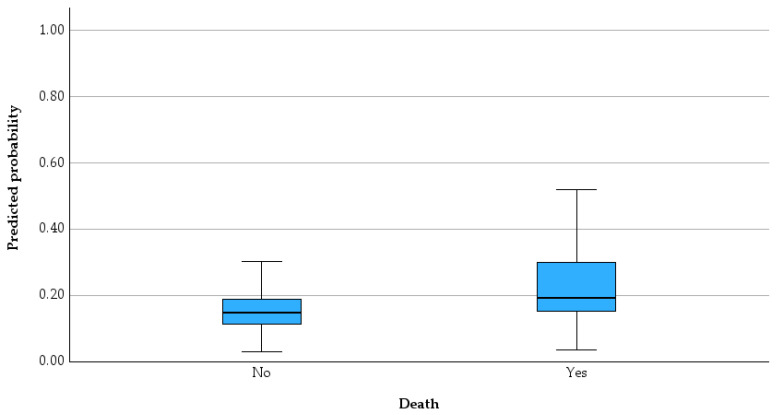
Observed versus predicted probabilities for outcome classification in the logistic regression model.

Figure 7 illustrates the distribution of observed outcomes (Yes/No) against the predicted probabilities generated by the logistic regression model, with a classification cut-off value of 0.50.

The model demonstrates that patients with higher PESI scores, elevated procalcitonin levels, and increased WBC counts have a significantly higher probability of experiencing the studied outcome (i.e., belonging to the “Yes” group), suggesting that both clinical severity and inflammatory status play a key role in risk stratification. Specifically, each incremental increase in PESI score, procalcitonin, and WBC is associated with a measurable rise in the odds of the outcome, supporting their utility as clinically relevant predictors.

In contrast, age and INR were not statistically significant, indicating that, within this cohort, they do not independently contribute to outcome prediction when adjusted for other variables.

From a practical standpoint, the model’s predicted probability plot shows that most low-risk patients (low predicted probabilities) are correctly classified as “No,” while high-risk patients cluster toward higher probabilities and are more frequently classified as “Yes.” However, the overlap observed in intermediate probability ranges highlights a subgroup of patients with uncertain risk, where clinical judgment remains essential. The distribution patterns of key predictors and their relationship with outcomes are further illustrated in the supplementary histograms and Q–Q plots (Figures S5–S12), supporting the observed statistical findings.

## 4. Discussion

The present study evaluated the prognostic value of commonly used clinical risk scores and laboratory parameters in patients with pulmonary embolism. Our results demonstrated that higher PESI, WELLS, PADUA, and IMPROVE scores were significantly associated with in-hospital mortality, and that inflammatory markers such as procalcitonin and WBC also showed independent associations with death in multivariate analysis.

Similarly to our findings, the prognostic value of the PESI score has been widely reported in the literature. A study conducted by Jara-Palomares et al. reported that the PESI score provides effective in-hospital mortality risk stratification in pulmonary embolism, with high sensitivity for identifying low-risk patients and predicting 30-day mortality [25]. Likewise, the study led by De Wit et al. highlighted that the current European Society of Cardiology guidelines recommend the use of validated clinical prognostic tools such as PESI or simplified PESI for in-hospital mortality risk stratification in acute pulmonary embolism [26].

Consistent with our results showing an association between the WELLS score and mortality risk, the clinical application of the WELLS score for pulmonary embolism probability assessment has been extensively described, with established thresholds for low, moderate, and high probability of disease. Similar observations were reported in the study of Vyas et al., where clinical prediction rules were shown to assist in stratifying thromboembolic risk and guiding diagnostic evaluation [27].

Our findings regarding the prognostic relevance of the IMPROVE score are also consistent with previous research. As described by Spyropoulos et al., the IMPROVE score has been widely used for venous thromboembolism risk assessment in hospitalized patients and has demonstrated utility in identifying individuals at increased thrombotic risk [28].

Comparable findings regarding the use of clinical decision scores for risk assessment in pulmonary embolism were reported by Zhou et al., where the authors emphasized the importance of validating prediction models and integrating clinical decision tools into patient risk stratification strategies [29].

While the PESI score provides a validated estimate of mortality risk based on clinical parameters, it does not fully capture the underlying inflammatory and metabolic responses associated with disease severity [30]. The addition of biomarkers such as procalcitonin and white blood cell count may reflect systemic inflammatory activation and tissue injury [31].

The presence of COVID-19 infection in 23.2% of patients may have influenced both laboratory parameters and clinical outcomes. COVID-19 is known to induce a pro-inflammatory and prothrombotic state, which can exacerbate endothelial dysfunction and coagulation abnormalities [32]. This often leads to elevated inflammatory markers, including WBC and procalcitonin, and contributes to increased mortality risk [33]. Therefore, the coexistence of COVID-19 infection represents an important confounding factor that should be considered when interpreting the results.

The significant association between several laboratory parameters and mortality likely reflects the systemic impact of pulmonary embolism [34]. Elevated inflammatory markers are indicators of a more severe physiological response, while abnormalities in coagulation and organ function markers suggest advanced disease and multiorgan involvement [35].

White blood cell count emerged as an independent predictor of mortality, likely reflecting the degree of systemic inflammatory response. In the context of pulmonary embolism, leukocytosis may be associated with endothelial activation, cytokine release, and increased thrombotic burden [36]. Elevated liver enzymes, including AST, ALT, and GGT, reflect hepatic congestion secondary to right ventricular dysfunction in acute pulmonary embolism [37].

The high use of fondaparinux in this cohort reflects local clinical practice patterns [38]. Although anticoagulant therapy is essential in the management of pulmonary embolism, this study was not designed to compare treatment efficacy between different agents. Therefore, no direct conclusions can be drawn regarding the impact of specific anticoagulants on mortality outcomes.

This study has several limitations that should be acknowledged. In particular, the comparison between survivors and non-survivors was based on real-world clinical data without the application of propensity score matching, which may limit the comparability of the groups and allow for residual confounding. Although we attempted to mitigate this effect by performing multivariable logistic regression analysis, including key variables associated with in-hospital mortality, unmeasured confounders cannot be entirely excluded. Therefore, the findings should be interpreted with caution, and future prospective studies incorporating matched cohorts are warranted to validate these results. Additionally, the inclusion of patients with COVID-19 infection may have affected both laboratory parameters and mortality risk.

## 5. Conclusions

This study evaluated the prognostic value of commonly used clinical risk scores and laboratory markers in patients diagnosed with pulmonary embolism. The study population consisted of 538 patients, with an in-hospital mortality rate of 18.4%. Baseline analysis demonstrated a balanced sex distribution and a predominance of patients from urban areas. Thrombus localization was most frequently observed in the lobar and segmental pulmonary arteries, followed by the main pulmonary artery.

Comparative statistical analyses showed significant differences between survivors and non-survivors for all evaluated clinical scores. The PESI, WELLS, PADUA, and IMPROVE scores were significantly higher among patients who died during hospitalization. ROC curve analysis demonstrated varying predictive performance among the four clinical scores, with PESI showing the most pronounced discriminatory capacity for mortality.

Laboratory analyses identified several biomarkers significantly associated with mortality. White blood cell count, neutrophils, lymphocytes, platelet count, total bilirubin, AST, ALT, GGT, procalcitonin, and INR differed significantly between survivors and non-survivors. Multivariate logistic regression analysis demonstrated that the PESI score, procalcitonin, and WBC were independently associated with mortality, while age and INR were not significant predictors in the final model. The results demonstrate that clinical risk scores and selected laboratory markers provide measurable prognostic information in patients with pulmonary embolism.

The integration of clinical scoring systems with selected biomarkers reflecting inflammatory and coagulation pathways may improve mortality prediction and support more precise risk stratification in the acute setting. However, these findings are limited to in-hospital outcomes and should be interpreted within the context of a retrospective study design. Further prospective studies are warranted to validate these results and to determine the clinical utility of combined risk models in optimizing patient management.

## Figures and Tables

**Table 1:** Baseline demographic and clinical characteristics of the study population.

		Count	Table N%	95.0% Lower CL	95.0% Upper CL
Sex	Male	265	49.3%	45.0%	53.5%
	Female	273	50.7%	46.5%	55.0%
Urban	No	223	41.4%	37.3%	45.6%
	Yes	315	58.6%	54.4%	62.7%
In-hospital mortality	No	439	81.6%	78.1%	84.7%
	Yes	99	18.4%	15.3%	21.9%
Thrombus location	Main artery	138	25.6%	22.2%	29.6%
	Lobar artery	159	29.5%	25.9%	33.6%
	Segmental artery	156	29%	25.4%	33.1%
	Subsegmental artery	20	3.71%	2.4%	5.6%
	Truncus pulmonalis	21	3.9%	2.5%	5.8%
	Other localizations	44	8.1%	5.8%	10.3%
Antiplatelet therapy	No	419	77.8%	74.1%	81.2%
	Yes	119	22.11%	18.8%	25.9%
Cardiac failure (NYHA class)	0	184	34.2%	30.3%	38.3%
	1	62	11.5%	9.0%	14.4%
	2	195	36.2%	32.3%	40.4%
	3	84	15.6%	12.7%	18.9%
	4	13	2.4%	1.4%	4.0%
Anticoagulant therapy	No	380	70.6%	66.7%	74.4%
	Yes	158	29.4%	25.6%	33.3%
Pulmonary hypertension	No	304	56.5%	52.3%	60.7%
	Yes	234	43.5%	39.3%	47.7%
Diabetes	No	415	77.1%	73.4%	80.5%
	Yes	123	22.9%	19.5%	26.6%
Cancer	No	431	80.1%	76.6%	83.3%
	Yes	107	19.9%	16.7%	23.4%
Obesity	No	370	68.8%	64.8%	72.6%
	Yes	168	31.2%	27.4%	35.2%
Sepsis	No	463	86.1%	82.9%	88.8%
	Yes	75	13.9%	11.2%	17.1%
Associated infections	No	296	55%	51.1%	59.5%
	Yes	242	44.98%	40.5%	48.9%
Alteplase	No	490	91.07%	88.6%	93.4%
	Yes	48	8.9%	6.6%	11.4%
Nadroparin	No	479	89.03%	86.1%	91.4%
	Yes	59	10.96%	8.6%	13.9%
Unfractionated heparin	No	479	89.0%	86.2%	91.5%
	Yes	59	11.0%	8.5%	13.8%
Vitamin K antagonist	No	501	93.1%	90.8%	95.0%
	Yes	37	6.9%	5.0%	9.2%
Enoxaparin	No	342	63.6%	59.4%	67.6%
	Yes	196	36.4%	32.4%	40.6%
Fondaparinux	No	120	22.3%	18.9%	26.0%
	Yes	418	77.7%	74.0%	81.1%
Comorbidities	No	93	17.3%	14.3%	20.7%
	Yes	445	82.7%	79.3%	85.7%
Intubated	No	444	82.5%	79.1%	85.6%
	Yes	94	17.5%	14.4%	20.9%
CPAP	No	469	87.2%	84.2%	89.8%
	Yes	69	12.8%	10.2%	15.8%
COVID-19	No	413	76.8%	73.1%	80.2%
	Yes	125	23.2%	19.8%	26.9%

NYHA, New York Heart Association functional classification; CPAP, continuous positive airway pressure; COVID-19, coronavirus disease 2019. NYHA class 0 indicates absence of symptoms of heart failure.

**Table 2:** Laboratory parameters and clinical risk scores in the study population.

	Mean	SD	95.0% Lower CL	95.0% Upper CL	Median	Maximum	Minimum
Age	69	14	67	70	70	95	20
Biologic values							
WBC (10^9^/L)	10	5	9	10	10	38	1
Neutrophils (10^9^/L)	10	12	9	11	8	92	1
Lymphocytes (10^9^/L)	2	2	2	2	2	41	0
PLT (10^9^/L)	231	97	223	240	217	622	10
Creatinine (mg/dL)	1.36	4.06	1.02	1.71	1.06	94.00	0.18
Total bilirubin (mg/dL)	0.77	0.78	0.70	0.83	0.60	11.00	0.10
AST (U/L)	67	233	48	87	31	3420	8
ALT (U/L)	67	223	48	86	32	3730	4
GGT (U/L)	71	72	65	77	49	783	0
Procalcitonin (ng/mL)	1.17	3.58	0.87	1.47	0.25	58.12	0.00
d-Dimers (mg/L)	10	25	8	12	5	528	0
aPTT (seconds)	26.0	8.4	25.3	26.7	24.6	113.0	11.0
INR	1.16	0.33	1.13	1.19	1.10	5.70	0.11
Na (mmol/L)	139	5	138	139	139	166	115
K (mmol/L)	4.1	0.6	4.1	4.2	4.1	6.5	2.0
Scores							
PESI	110	33	107	113	110	257	7
WELLS	1.8	2.0	1.7	2.0	1.5	9.0	0
PADUA	3	2	3	4	3	11	0
IMPROVE	3	2	2	3	2	8	0

WBC, white blood cell count; Neutrophils, absolute neutrophil count; Lymphocytes, absolute lymphocyte count; PLT, platelet count; AST, aspartate aminotransferase; ALT, alanine aminotransferase; GGT, gamma-glutamyl transferase; INR, international normalized ratio; aPTT, activated partial thromboplastin time; Na, sodium; K, potassium; PESI, Pulmonary Embolism Severity Index; WELLS, WELLS score for pulmonary embolism probability; PADUA, PADUA prediction score; IMPROVE, International Medical Prevention Registry on Venous Thromboembolism score.

**Table 3:** Baseline demographic, clinical characteristics, and risk scores stratified by in-hospital mortality.

		Death					
		No			Yes		
		Count	N%	Mean	Count	N%	Mean
Sex	Male	223	84.2%		42	15.8%	
	Female	216	79.1%		57	20.9%	
Urban	No	167	74.9%		56	25.1%	
	Yes	272	86.3%		43	13.7%	
Thrombus location	Main artery	116	84.1%		22	15.9%	
	Lobar artery	132	83.0%		27	17.0%	
	Segmental artery	119	76.3%		37	23.7%	
	Subsegmental artery	18	90.0%		2	10.0%	
	Truncus pulmonalis	18	85.7%		3	14.3%	
	Other localizations	35	79.5%		9	20.4%	
PESI				106			126
WELLS				1.7			2.5
PADUA				3			4
IMPROVE				2			4
Antiplatelet therapy	No	338	80.6%		81	19.3%	
	Yes	101	84.9%		18	15.1%	
Cardiac failure	0	153	83.2%		31	16.8%	
	1	44	71.0%		18	29.0%	
	2	173	88.7%		22	11.3%	
	3	62	73.8%		22	26.2%	
	4	7	53.8%		6	46.2%	
Anticoagulant therapy	No	321	84.5%		59	15.5%	
	Yes	118	74.7%		40	25.3%	
PH	No	229	75.3%		75	24.7%	
	Yes	210	89.7%		24	10.3%	
Diabetes	No	333	80.2%		82	19.8%	
	Yes	106	86.2%		17	13.8%	
Cancer	No	353	81.9%		78	18.1%	
	Yes	86	80.4%		21	19.6%	
Obesity	No	287	77.6%		83	22.4%	
	Yes	152	90.5%		16	9.5%	
Sepsis	No	409	88.3%		54	11.7%	
	Yes	30	40.0%		45	60.0%	
Associated infections	No	250	84.5%		46	15.5%	
	Yes	186	77.8%		56	23.1%	
COVID-19	No	350	84.7%		63	15.3%	
	Yes	89	71.2%		36	28.8%	
CPAP	No	402	85.7%		67	14.3%	
	Yes	37	53.6%		32	46.4%	
Intubated	No	391	88.1%		53	11.9%	
	Yes	48	51.1%		46	48.9%	

PESI, Pulmonary Embolism Severity Index; WELLS, WELLS score for pulmonary embolism probability; PADUA, PADUA prediction score; IMPROVE, International Medical Prevention Registry on Venous Thromboembolism score; PH, pulmonary hypertension; CPAP, continuous positive airway pressure; COVID-19, coronavirus disease 2019.

**Table 4:** Comparison of age and clinical risk scores between survivors and non-survivors.

		Levene’s Test for Equality of Variances		*t*-Test for Equality of Means			
		Sig.	Significance	Mean Difference	Std. Error Difference	95% Confidence Interval of the Difference	
			Two-Sided *p*			Lower	Upper
Age	Equal variances assumed	0.921	*p* < 0.019	−3.725	1.577	−6.823	−0.628
PESI	Equal variances not assumed		*p* < 0.001	−19.233	4.076	−27.298	−11.169
WELLS	Equal variances not assumed		*p* < 0.001	−0.8568	0.2542	−1.3599	−0.3537
PADUA	Equal variances not assumed		*p* < 0.001	−1.389	0.288	−1.959	−0.820
IMPROVE	Equal variances assumed	0.128	*p* < 0.001	−1.191	0.203	−1.590	−0.791

**Table 5:** Comparison of laboratory parameters between survivors and non-survivors using the Mann–Whitney U test.

Variable	Mann–Whitney U	Wilcoxon W	Z	*p*
Significant				
WBC (10^9^/L)	14,784.000	110,925.000	−4.947	<0.001
Neutrophils (10^9^/L)	13,835.500	109,538.500	−5.603	<0.001
Lymphocytes (10^9^/L)	16,037.500	20,987.500	−4.049	<0.001
PLT (10^9^/L)	18,678.000	23,628.000	−2.154	0.031
Total bilirubin (mg/dL)	17,787.000	113,928.000	−2.794	0.005
AST (U/L)	15,843.000	111,984.000	−4.188	<0.001
ALT (U/L)	18,904.000	115,045.000	−1.992	0.046
GGT (U/L)	17,647.500	113,788.500	−2.894	0.004
Procalcitonin (ng/mL)	15,318.000	111,459.000	−4.502	<0.001
INR	16,345.000	112,486.000	−3.830	<0.001
Not significant				
Creatinine (mg/dL)	20,507.000	116,648.000	−0.842	0.400
d-Dimer (mg/L)	21,076.000	117,217.000	−0.434	0.664
aPTT (seconds)	20,564.000	25,514.000	−0.801	0.423
Sodium (mmol/L)	20,003.000	116,144.000	−1.207	0.228
Potassium (mmol/L)	20,657.500	116,798.500	−0.735	0.462

Grouping Variable: Death; WBC, white blood cell count; Neutrophils, absolute neutrophil count; Lymphocytes, absolute lymphocyte count; PLT, platelet count; AST, aspartate aminotransferase; ALT, alanine aminotransferase; GGT, gamma-glutamyl transferase; INR, international normalized ratio; aPTT, activated partial thromboplastin time.

**Table 6:** Multivariate logistic regression analysis identifying independent predictors of in-hospital mortality.

		B	S.E.	Wald	df	Sig.	Exp(B)	95% C.I. for Exp(B)	
								Lower	Upper
Step 1 ^a^	Age	0.012	0.010	1.560	1	0.212	1.012	0.993	1.032
	PESI	0.013	0.004	11.438	1	<0.001	1.013	1.006	1.021
	Procalcitonin	0.101	0.038	7.279	1	0.007	1.107	1.028	1.191
	WBC	0.097	0.021	20.339	1	<0.001	1.102	1.056	1.149
	INR	0.141	0.310	0.207	1	0.649	1.152	0.627	2.113
	Constant	−5.214	0.803	42.209	1	<0.001	0.005		

a. Variable(s) entered on step 1: Age, PESI, Procalcitonin, WBC, INR.

## Data Availability

Data is available on request to the corresponding author.

## Supplementary Materials

The following supporting information can be downloaded at: https://www.mdpi.com/article/10.3390/jcm15083141/s1. Figure S1: Precision–Recall curve for PESI score; Figure S2: Precision–Recall curve for WELLS score; Figure S3: Precision–Recall curve for PADUA score; Figure S4: Precision–Recall curve for IMPROVE score; Table S1: Tests of normality; Figure S5: Age distribution in the non-fatal group; Figure S6: Age distribution in the fatal group; Figure S7: Normal Q–Q plot of age (non-fatal group); Figure S8: Normal Q–Q plot of age (fatal group); Figure S9: Distribution of PESI score (fatal group); Figure S10: Distribution of WELLS score (fatal group); Figure S11: Distribution of PADUA score (fatal group); Figure S12: Distribution of IMPROVE score (fatal group).
