# Supplementary material for: Risk Stratification in Pulmonary Embolism: Prognostic Value of PESI, WELLS, PADUA, and IMPROVE Scores in Relation to Laboratory Markers
Source: J Clin Med. 2026 Apr 20;15(8):3141. doi: 10.3390/jcm15083141 (PMC13118125; doi:10.3390/jcm15083141)

SUPPLEMENTARY MATERIAL

Figure S1. Precision-Recall Curve – PESI score

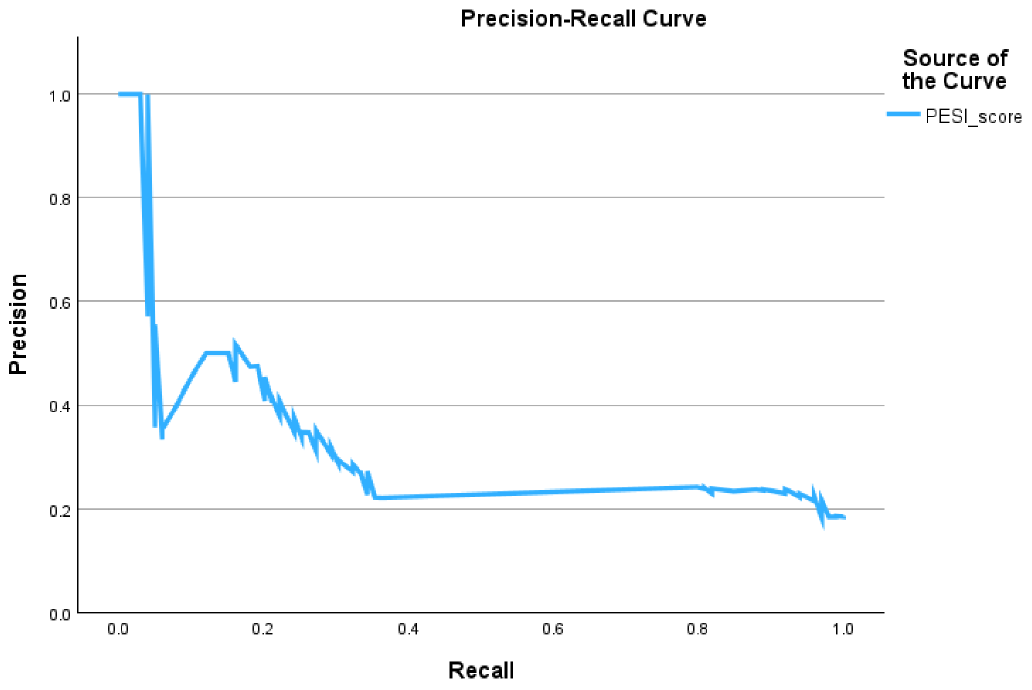

Figure S2. Precision-Recall Curve – WELLS score

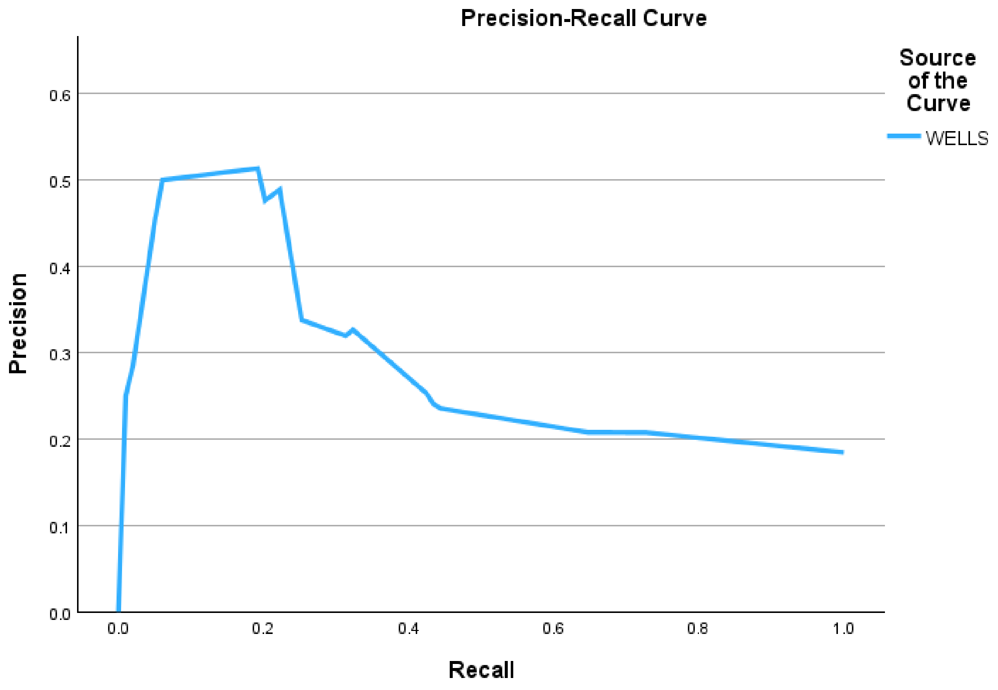

Figure S3. Precision-Recall Curve – PADUA score

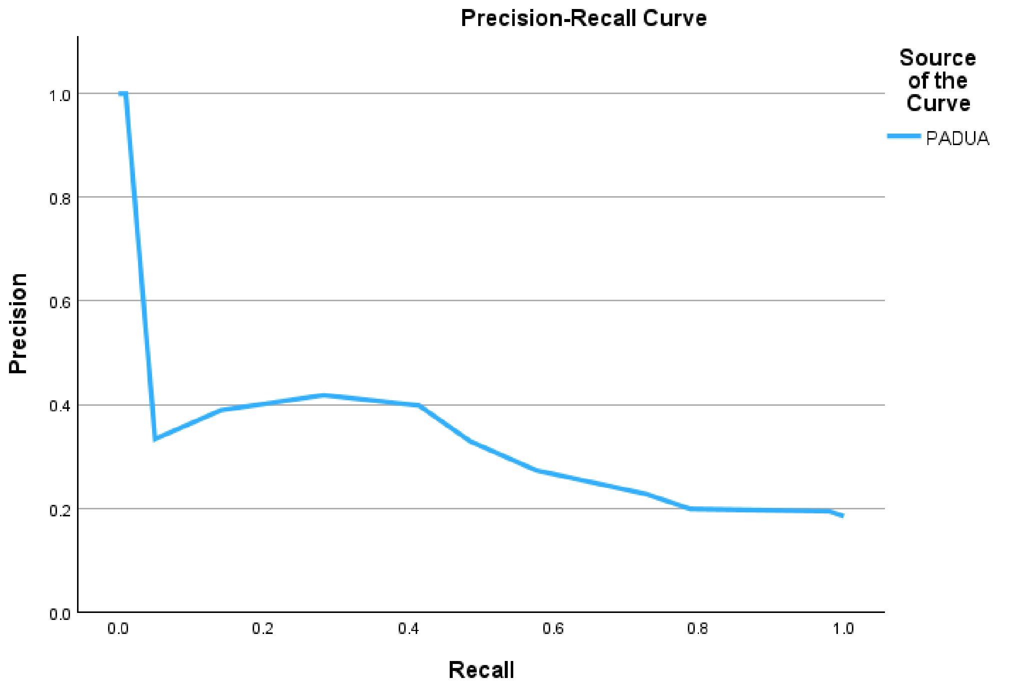

Figure S4. Precision-Recall Curve – IMPROVE score

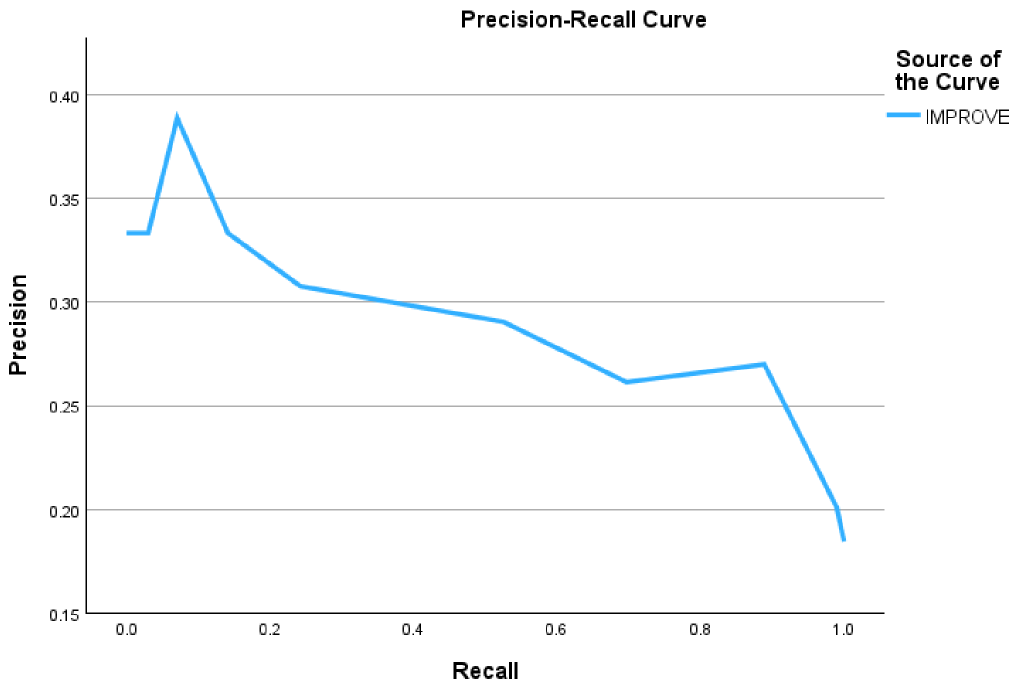

Table S1: Tests of Normality

		Kolmogorov-Smirnov[a]			Shapiro-Wilk		
	Death	Statistic	df	Sig.	Statistic	df	Sig.
Age	No	.078	439	<.001	.964	439	<.001
	Yes	.090	99	.048	.931	99	<.001
PESI	No	.165	439	<.001	.946	439	<.001
	Yes	.298	99	<.001	.840	99	<.001
WELLS	No	.212	439	<.001	.841	439	<.001
	Yes	.223	99	<.001	.876	99	<.001
PADUA	No	.167	439	<.001	.925	439	<.001
	Yes	.133	99	<.001	.938	99	<.001
IMPROVE	No	.232	439	<.001	.899	439	<.001
	Yes	.165	99	<.001	.947	99	<.001
NTproBNP	No	.291	439	<.001	.559	439	<.001
	Yes	.346	99	<.001	.408	99	<.001
WBC	No	.073	439	<.001	.960	439	<.001
	Yes	.045	99	.200*	.974	99	.044
Neutrophils	No	.193	438	<.001	.488	438	<.001
	Yes	.306	99	<.001	.603	99	<.001
Lymphocytes	No	.138	439	<.001	.627	439	<.001
	Yes	.331	99	<.001	.354	99	<.001
PLT	No	.074	439	<.001	.951	439	<.001
	Yes	.128	99	<.001	.940	99	<.001
Creatinine	No	.417	439	<.001	.068	439	<.001
	Yes	.149	99	<.001	.862	99	<.001
Total bilirubin	No	.200	439	<.001	.516	439	<.001
	Yes	.267	99	<.001	.442	99	<.001
AST	No	.405	439	<.001	.141	439	<.001
	Yes	.382	99	<.001	.221	99	<.001
ALT	No	.396	439	<.001	.146	439	<.001
	Yes	.372	99	<.001	.241	99	<.001
GGT	No	.181	439	<.001	.723	439	<.001
	Yes	.214	99	<.001	.690	99	<.001
NA	No	.081	439	<.001	.966	439	<.001
	Yes	.150	99	<.001	.936	99	<.001
K	No	.093	439	<.001	.980	439	<.001
	Yes	.115	99	.002	.967	99	.013
Procalcitonin	No	.392	439	<.001	.406	439	<.001
	Yes	.364	98	<.001	.344	98	<.001
D dimers	No	.364	439	<.001	.197	439	<.001
	Yes	.233	99	<.001	.621	99	<.001
aPTT	No	.224	439	<.001	.528	439	<.001
	Yes	.207	99	<.001	.721	99	<.001

INR	No	.226	439	<.001	.494	439	<.001
	Yes	.140	99	<.001	.823	99	<.001

*. This is a lower bound of the true significance.
a. Lilliefors Significance Correction

Figure S5. Age distribution of patients in the non-fatal (death = no) group

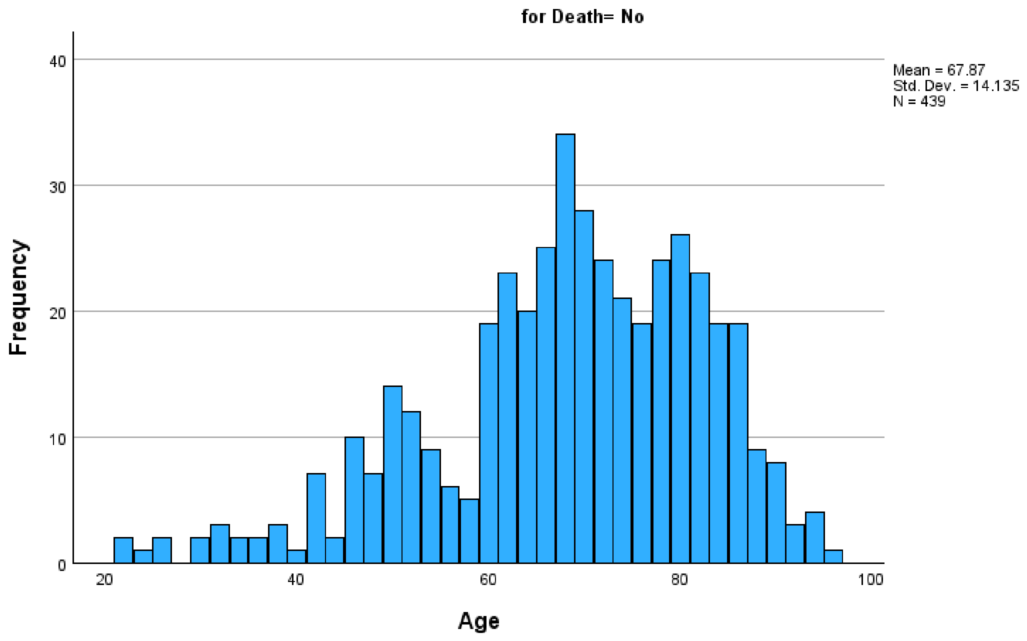

Figure S6. Age distribution of patients in the fatal (death = yes) group

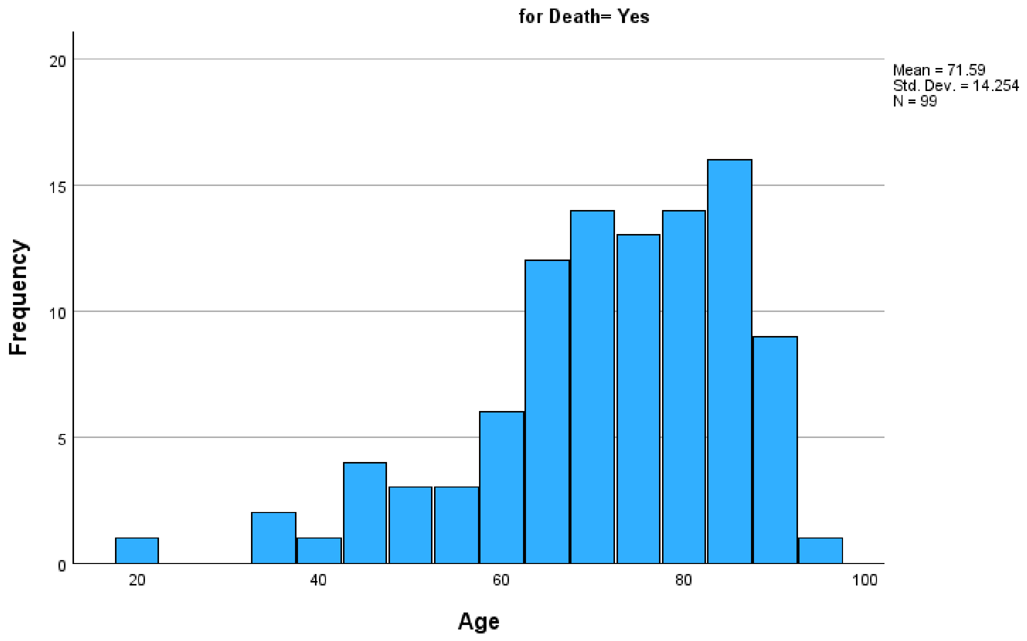

Figure S7. Normal Q–Q plot of age distribution in the non-fatal (death = no) group

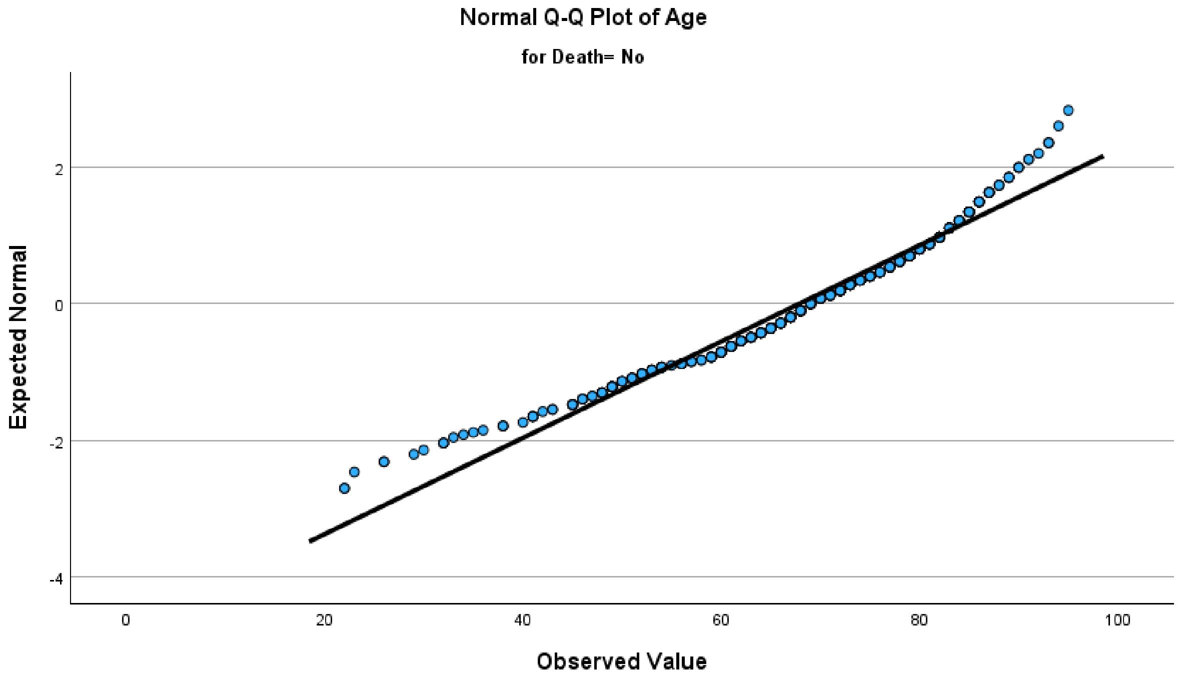

Figure S8. Normal Q–Q plot of age distribution in the fatal (death = yes) group

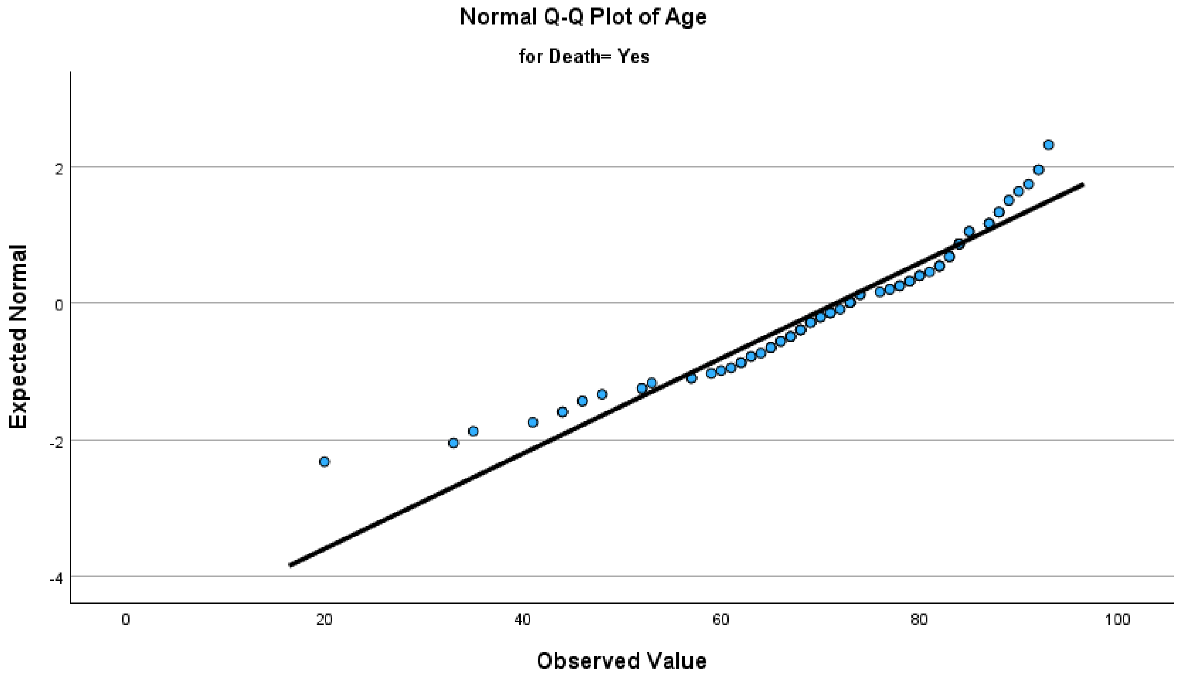

Figure S9. Distribution of PESI score in the fatal (death = yes) group

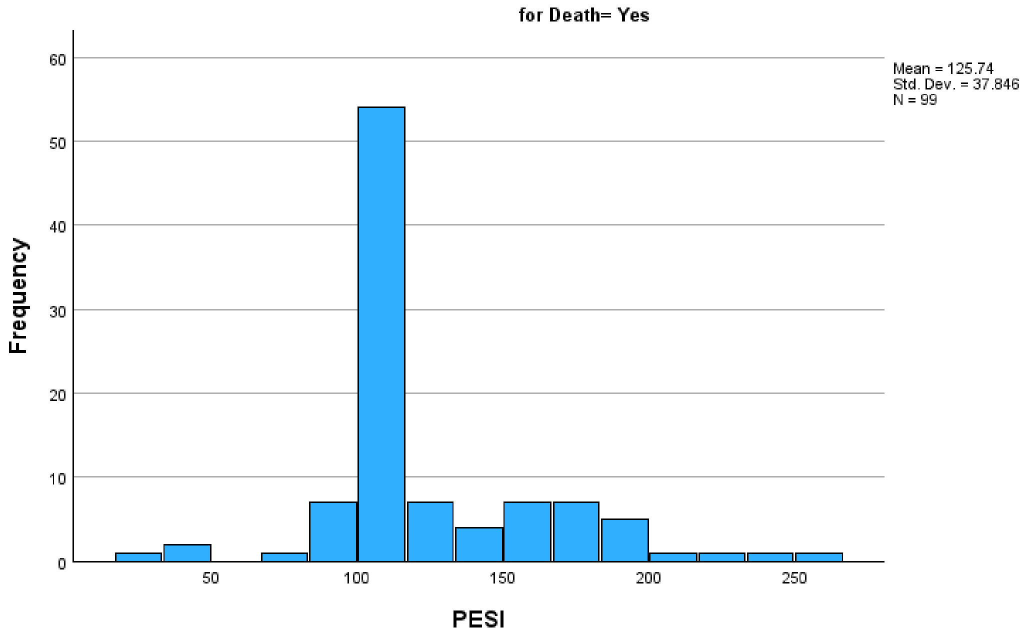

Figure S10. Distribution of Wells score in the fatal (death = yes) group

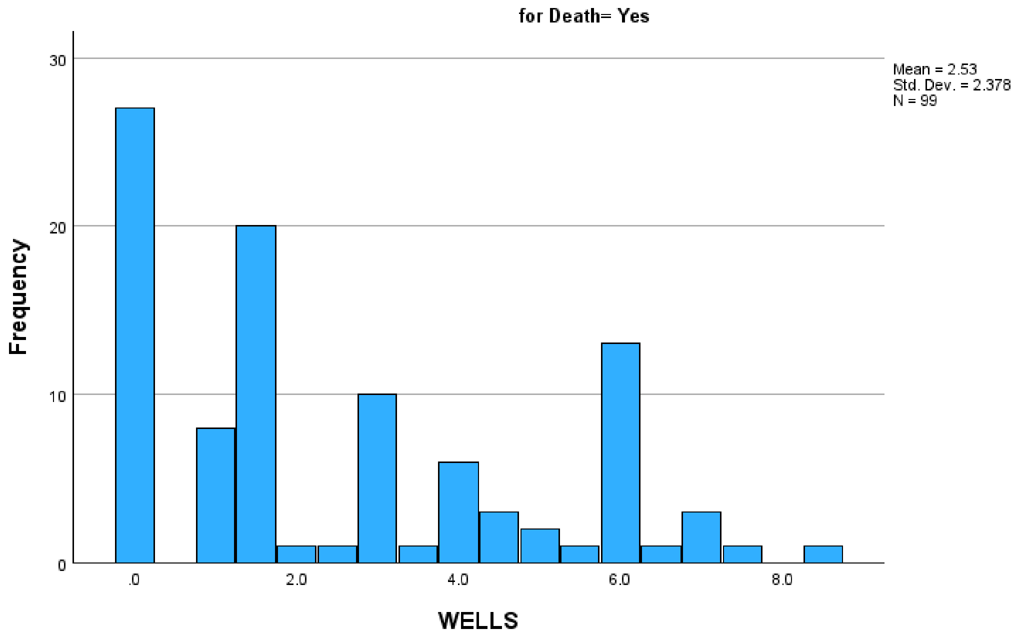

Figure S11. Distribution of PADUA score in the fatal (death = yes) group

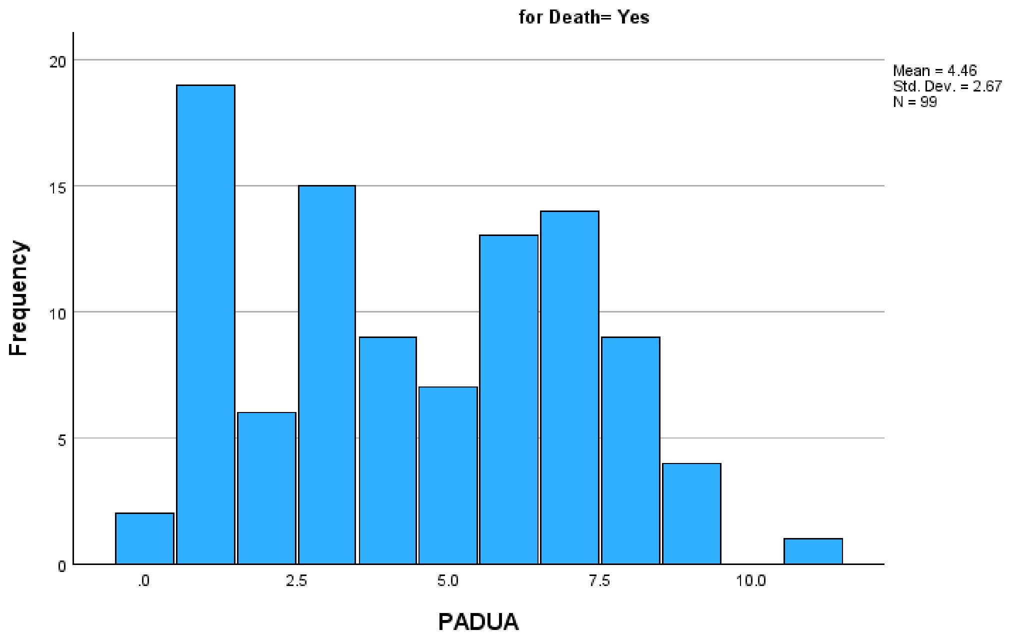

Figure S12. Distribution of IMPROVE score in the fatal (death = yes) group

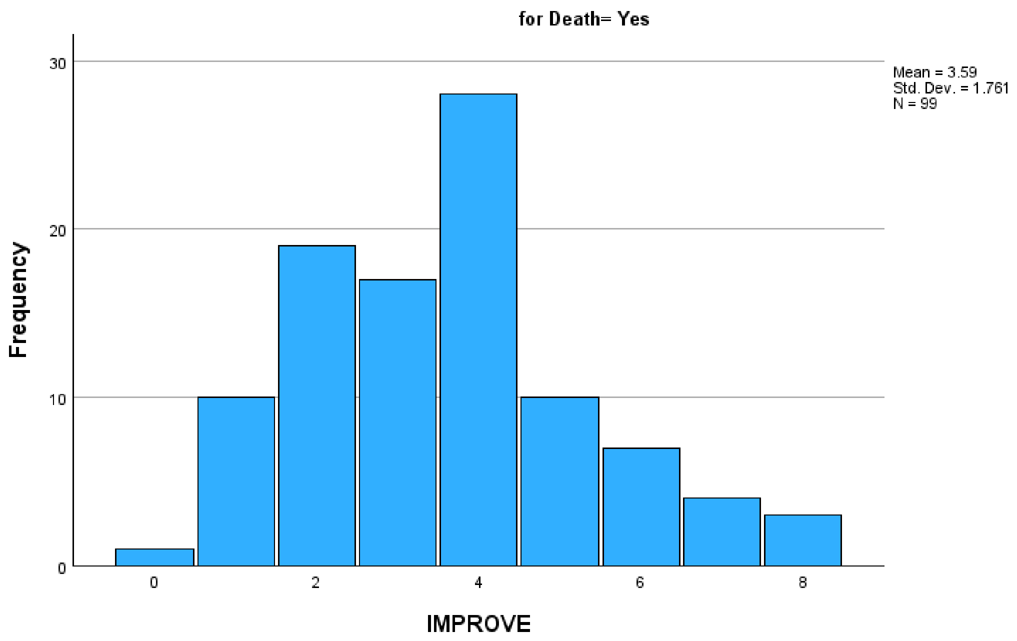

Supplement: Supplementary file 1 [file jcm-15-03141-s001.zip › jcm-4235128-supplementary.pdf]
